# Medication Adherence and Its Influencing Factors in Postoperative Thyroid Cancer Patients: A Cross-Sectional Study

**DOI:** 10.1155/nrp/6943237

**Published:** 2025-04-28

**Authors:** Wen Li, Luxin Yu, Hui Zheng, Kim Geok Soh, Kim Lam Soh

**Affiliations:** ^1^Gastrointestinal Surgery, Yantai Yuhuangding Hospital, Yantai, Shandong, China; ^2^Nursing Department, Yantai Yuhuangding Hospital, Yantai, Shandong, China; ^3^Department of Sport Studies, Faculty of Educational Studies, University Putra Malaysia, Serdang, Selangor Darul Ehsan, Malaysia; ^4^Department of Nursing, Faculty of Medicine and Health Sciences, University Putra Malaysia, Serdang, Selangor Darul Ehsan, Malaysia

## Abstract

**Background:** Medication adherence is critical for the recovery of patients after thyroid cancer surgery. Therefore, this study investigated the current medication adherence status in Chinese postoperative thyroid cancer patients and analysed the influencing factors.

**Methods:** A cross-sectional descriptive survey of postoperative thyroid cancer patients at a tertiary-level hospital in Shandong Province, China, from January to June 2019 was conducted, and 266 postoperative thyroid cancer patients were included. The patient's medication adherence was assessed with the Chinese version of the Morrissey Medication Adherence Scale, and the related influencing factors were measured with the General Information Questionnaire and the Rational Medication Self-Efficacy Scale. Finally, factors associated with medication adherence were explored using multiple linear regression analysis.

**Results:** The medication adherence for postoperative thyroid cancer patients is at an intermediate level with a score of (6.25 ± 1.57). Patients' rational medication self-efficacy was positively correlated with medication adherence, with a correlation coefficient (*r*) of 0.536 (*p* < 0.001). Multiple linear regression analyses revealed that education level, disease duration and self-efficacy for rational medication use were the main factors affecting medication adherence in postoperative thyroid cancer patients.

**Conclusion:** Improving patients' self-efficacy in rational medication and strengthening patients' health education are important ways to enhance patients' medication adherence.

## 1. Introduction

Thyroid cancer (TC) is a malignant tumour originating from the follicular epithelial or parafollicular epithelial cells of the thyroid gland, accounting for 1%–4% of all malignant tumours, and is the most common malignant tumour of the head and neck [1]. As the thyroid tumour grows, it gradually compresses the trachea, oesophagus and recurrent laryngeal nerve, leading to symptoms such as difficulty breathing, difficulty swallowing and hoarseness [2]. In recent years, the incidence of TC has continued to grow rapidly worldwide and is now the seventh most prevalent malignant tumour in the world [3]. In China, TC continues to grow at a rate of 20% per year, and the number of cases in 2020 was about 221,000, accounting for about 1/3 of the world's total number of TCs. The age-specific incidence rate of thyroid cancer in China has ranked third among all malignant tumours, posing a serious threat to the health of the Chinese population [4]. According to the report of Bray et al. [5], there were 466,000 new cases of TC in China in 2022, accounting for 56.76% of the total number of new cancer cases globally, with an age-standardized incidence rate of 24.6/100,000, higher than the global incidence rate of 9.1/100,000. Although the higher incidence of TC in China may be attributed to increased awareness of cancer prevention among the Chinese population, better medical care and more sophisticated testing equipment [6], it is undeniable that China has a high incidence of thyroid cancer. What is more, according to national cancer statistics released in 2022, the death rate from thyroid cancer is on a continuous upward trend, and the 5-year survival rate of 84.3% of Chinese thyroid cancer patients is much lower than that of the United States, with a gap of about 14% [5].

Currently, surgery is the preferred treatment of choice for TC, but postoperative patients still require lifelong thyroxine sodium tablets to maintain normal thyroid function [7]. Available studies showed that failure to take medication on time and following the dosage would lead to hypothyroidism and mucous oedema coma, increase the possibility of tumour recurrence and even endanger the patient's life [8, 9]. However, a study by Mols et al., 2020, showed that 52% of postoperative thyroid cancer patients were not adherent to their medication [10]. Therefore, it is essential to analyse and understand the factors affecting medication adherence for improving medication adherence and accelerating recovery in postoperative thyroid cancer patients. Accumulated data suggested that patients' medication adherence is influenced by many factors, such as the patient's knowledge, ability and self-efficacy [11–13]. Although China is more threatened by thyroid cancer than Western countries, Chinese researchers have not paid enough attention to medication adherence in patients with TC, and there is no research data on the factors affecting medication adherence in patients with thyroid cancer specifically. Therefore, this study assessed the current status of medication adherence in Chinese postoperative thyroid patients and analysed the main factors influencing medication adherence. The purpose of this study is to provide basic data for improving medication adherence and thus promoting postoperative recovery in Chinese patients with thyroid glands in China.

## 2. Methods

### 2.1. Study Design, Sample and Settings

This study was reported based on the STROBE checklist (cross-sectional studies), as detailed in Table A1. A cross-sectional study was conducted from January to June 2019 in a tertiary hospital in China. The following inclusion and exclusion criteria were applied in the recruitment process. Inclusion criteria were as follows: (1) postoperative patients with a pathological diagnosis of TC; (2) postoperative administration of liothyronine sodium tablets for life; (3) clear thinkers with the ability to understand and express themselves and (4) voluntary participation in this study. Exclusion criteria were as follows: (1) patients with cognitive impairment and (2) patients with other serious endocrine, circulatory, respiratory or other diseases. The total number of scale items in this study was 21, and the sample size was determined to be 10–20 times the number of questionnaire items [14]. Besides, considering the non-response phenomenon that occurred during the collection process, the sample size was increased by 5% and the final sample size was determined to be 273 cases. Before the survey, the researcher explained the purpose and significance of the study in detail to the participants, and the participants signed the informed consent form in an informed manner. Besides, patients were surveyed at routine visits 3–6 months after surgery, and questionnaires were completed by patients who were invited to complete them when they returned to the thyroid clinic for review after surgery with one questionnaire taking approximately 10–15 min to complete. The questionnaires were completed anonymously and collected on the spot.

### 2.2. Instruments

#### 2.2.1. General Information Questionnaire

This section consists of sociodemographic data (e.g., age, gender, education level, marital status, monthly income and residency style and medical payment method) and disease-related information (e.g., surgical approach, disease duration, complicated by other diseases and family medical history).

#### 2.2.2. Self-Efficacy for Appropriate Medication Use Scale (SEAMS)

The self-efficacy in medication-taking behaviour in patients was assessed by the SEAMS, developed by Risser et al. in 2007 [15] and Chinese-language translated by Dong et al. in 2015 [16]. The scale consists of 13 items in 2 dimensions: self-efficacy in uncertain situations (5 items) and self-efficacy in difficult situations (8 items). This is a 3-point Likert scale ranging from “not confident” to “very confident” on a scale of 1 to 3. The total score ranges from 13 to 39, with higher scores indicating greater self-efficacy for patients to adhere to rational medication use. The Cronbach's alpha coefficient for the scale was 0.934, the content validity index was 0.913 [16]. Our previous study showed that the Cronbach's alpha coefficient of the scale in postoperative thyroid cancer patients was 0.915, indicating that the scale has good reliability and validity [17].

#### 2.2.3. Eight-Item Morisky Medication Adherence Scale (MMAS-8)

The MMAS-8 has been widely used to measure medication adherence in different types of patients [12].

The scale consists of 8 items. Items 1–7 are answered dichotomously using “yes” or “no” answers and item 8 is responded to using a 5-point Likert scale. For scoring each “no” response is rated as 0 and each “yes” response is rated as 1. Item 5 is scored the opposite way, i.e., a “yes” response was scored as 0 and a “no” response was scored as 1. Item 8 was scored on a 5-point Likert scale, with “Never,” “Occasionally,” “Sometimes,” “Often” and “Always” scoring 1.0, 0.75, 0.50, 0.25 and 0 points, respectively, in that order. The scores for each question were summed to give a total score, with higher scores indicating better medication adherence. The scale was scored out of 8, with a score of < 6 indicating poor adherence, a score of ≥ 6 and < 8 indicating moderate adherence and a score of 8 indicating good adherence. The scale was introduced in China by domestic scholars in 2013, and Cronbach's alpha coefficient of the scale was 0.65 [18].

### 2.3. Data Analysis

The data were entered into the Excel database. Continuous variables were described by means and standard deviations, and categorical data were described by frequencies and percentages. One-way analyses were performed using independent samples *t*-test, ANOVA or Pearson's correlation analysis. Multivariate linear regression analysis was used to analyse the factors influencing medication adherence. The abovementioned analyses were completed using SPSS 27.0 and *p* < 0.05 was considered statistically significant.

### 2.4. Ethical Considerations

The study was approved by the Institutional Review Board of the hospital. All subjects volunteered to participate in the study and signed an informed consent form. The data including the subjects' personal information were handled confidentially.

## 3. Results

### 3.1. Participant Characteristics

As shown in Table 1, the age of 266 postoperative thyroid cancer patients ranged from 25 to 76 years with a mean age of (48.05 ± 11.69) years. Among them, 71.18% were female, 93.98% were married, 84.96% lived with their spouse and 72.55% had mainly thyroid lobectomy with isthmus as the main surgical procedure and the disease duration ranged from 2 weeks to 10 years.

### 3.2. The Current Status of Medication Adherence in Postoperative Thyroid Cancer Patients

As shown in Table 2, the total medication adherence score was (6.25 ± 1.57). Of these, 114 patients (42.86%) had poor adherence, 94 patients (35.34%) had moderate adherence and 58 patients (21.80%) had good adherence. For item 8, “How often do you have difficulty remembering to take all your medications?”, 100 (37.59%) responded “never,” 138 (51.88%) “occasionally” and 28 (10.53%) “sometimes.” The overall medication adherence of postoperative thyroid cancer patients was moderate for each item.

### 3.3. Correlation Between Rational Medication Self-Efficacy and Medication Adherence in Postoperative Thyroid Cancer Patients

A normality test on each variable of the postoperative thyroid cancer patient survey revealed that both variables conformed to a normal distribution and, therefore, correlation analyses were performed using Pearson correlation analysis. The results showed that rational medication self-efficacy and medication adherence in postoperative thyroid cancer patients were significantly positively correlated, with a correlation coefficient (*r*) of 0.536 (*p* < 0.001).

### 3.4. A Multifactorial Analysis of Factors Affecting Medication Adherence in Postoperative Thyroid Cancer Patients

The results of the one-way analysis described above (see Table 3 for details) showed that five variables in the general data affected medication adherence scores of postoperative thyroid cancer patients. Besides, self-efficacy for rational medication use was associated with medication adherence. In this study, the dependent variable was medication adherence score, and multiple linear regression analyses were performed on the independent variables that were significant in the univariate analyses and the variables that were correlated with medication adherence. The results showed that disease duration (*β = *−0.197, *p*=0.001), education level (*β = *0.054, *p*=0.040) and self-efficacy for rational medication use (*β = *0.458, *p* < 0.001) were three factors affecting medication adherence among postoperative thyroid cancer patients, and three variables mentioned above explained 33.9% of the variance, as detailed in Table 4.

## 4. Discussion

### 4.1. The Medication Adherence of Postoperative Thyroid Cancer Patients

In the present study, the total medication adherence score of patients with thyroid cancer was at an intermediate level with a total score of (6.25 ± 1.57), which was lower than that reported by Alhemedi et al. [19] in Jordan but higher than the finding of Liu et al. [20] in China. Although patients need to take medication for a long period after surgery, patients' out-of-pocket medication costs decreased with the improvement of the reimbursement rate of health insurance, which reduced the financial burden of patients to a certain extent [21]. Besides, patients' awareness of taking medication gradually increases as their knowledge of the disease rises. Previous studies showed that patients attach great importance to obtaining information about cancer treatment and survival rates, and women and young patients, in particular, are more eager to get information and support [22, 23]. Furthermore, in this study, 42.86% of the patients had poor medication adherence after thyroid cancer surgery and only 21.8% had good medication adherence. Of these patients, 36.84% reported that they sometimes forgot to take their medication and 35.34% forgot to carry their medication with them when they were away or not at home, which is higher than the results of the study by Pelin et al. [13]. After thyroid cancer surgery, patients usually need to take thyroid hormone replacement therapy for a long period to maintain the level of thyroid hormones in the body. Irregularity or forgetting to take the medication may lead to fluctuations in hormone levels, affecting the patient's metabolism and recovery. This is especially common when patients are busy or unfocused in their daily lives. To address this issue, doctors and healthcare teams can help patients establish regular medication habits through reminder services, mobile apps or the help of family members. For example, medication record cards can be issued to patients, and small on-the-go pillboxes can be designed to remind patients to take their medication on time. Besides, the situation of patients forgetting to take their medication when they go out or are not at home also needs to be taken seriously, which may lead to interruption of medication taking and thus affect the effectiveness of treatment. To combat this issue, patients can consider keeping a small box with one or 2 days' worth of medication in their bag or purse for emergencies or family members may remind patients to carry their medication with them before they go out.

### 4.2. The Factors Influencing Medication Adherence of Postoperative Thyroid Cancer Patients

Our study showed that education level was one of the factors affecting medication adherence in postoperative thyroid cancer patients, and the higher the education level, the better the medication adherence. In general, better-educated patients usually have greater health awareness and self-management skills and are better able to understand the importance of their doctors' advice on adherence to medication and the health benefits of maintaining stable thyroid hormone levels over time. As a result, these patients may be more willing to take their medications on time and be able to take self-management measures, such as setting reminders and regularly checking for remaining medication, to ensure adherence [24]. However, less literate patients may face challenges in understanding and accepting medical advice. They may have a poor understanding of the need to take medication and the effects of hormone levels and thus have lower medication adherence. Moreover, language barriers or difficulty understanding medical terminology may make it more difficult for them to take their medications correctly. This requires the healthcare team to be more patient and attentive in their communication, helping them to understand the importance of treatment through clear and simple language and educational tools and providing effective medication management advice. Therefore, future studies should focus on patients with low education level, strengthen their education of disease-related knowledge through multiple channels, pay attention to patients' feedback, analyse and adjust the content of the guidance promptly and make regular return visits to help patients solve their postoperative problems.

Besides, our study showed that disease duration was another factor influencing medication adherence in patients with postoperative thyroid cancer; the longer the disease duration, the worse the medication adherence, which is consistent with the findings of Su et al. [25]. In the early postoperative period, patients and their families pay more attention to the prognosis of the disease, and they have higher adherence to taking medication and can strictly follow medical advice. However, as time passes and the patient's condition is under control or improves, the coordination between taking medication on time and life events may be contradictory, which leads to a gradual weakening of the patient's medication adherence [26, 27]. Besides, some patients may feel that they have “become a good doctor after being sick for a long time” and reduce or stop taking their medication without informing their doctor. Therefore, healthcare teams need to remind patients of the importance of ongoing medication and monitor their medication adherence through regular follow-up appointments and personalised health education.

Moreover, in this study, rational medication self-efficacy is another influencing factor of medication adherence in postoperative thyroid cancer patients, which is positively correlated with medication adherence. Rational medication self-efficacy refers to a patient's confidence and ability to effectively manage and take medications on time [24]. Previous studies showed that patients with higher self-efficacy are more able to overcome barriers and maintain stable medication management habits, thus improving treatment outcomes and prognosis [24, 28]. Besides, patients with higher self-efficacy pursue challenging goals and are able to work tirelessly and persevere in the face of difficulties and challenges [24], i.e., patients with higher self-efficacy are more likely to develop the ability to overcome difficulties and actively cooperate with their doctors' treatment. However, patients with low rational medication self-efficacy may face greater medication adherence challenges in adhering to their medications [29]. They may lack confidence in their treatment regimen and medication management, feel that they are unable to effectively manage their medications or be sceptical about the efficacy of their medications. Studies have shown that 67% of the patients are discharged back to the community [30], thus increasing the need for high levels of medication self-efficacy in daily life. In clinical practice, healthcare teams can improve medication adherence by enhancing patients' rational medication self-efficacy, which includes helping patients understand the importance of treatment and the mechanism of action of medications through education and presentation to increase their confidence in their treatment regimen. Besides, the healthcare team can work with the patient to develop a personalized medication management plan that provides support and advice based on the patient's lifestyle and specific needs. For example, the use of mobile apps with reminder functions, regular follow-up appointments to monitor the effectiveness of treatment and timely adjustments to drug dosages or treatment regimens.

### 4.3. Limitations of the Study

The first limitation of our study is that the study participants were all from the same tertiary hospital, which is not representative of the wider population. Second, participants' medication adherence was assessed through self-reported questions rather than any objective indicators, which may also lead to bias. In the future, the sample size should be further expanded and from different organisations to increase the generalisability and reliability of the findings.

## 5. Conclusions

The medication adherence for postoperative thyroid cancer patients is at an intermediate level. Education level, disease duration and self-efficacy for rational medication use are the three factors that affect patients' medication adherence. Nurses can further improve patients' medication adherence by improving patients' self-efficacy for rational medication use and enhancing patients' health education.

## Figures and Tables

**Table 1:** Participants' characteristics and supervision parameters (*N* = 266).

Projects	Response options	Number of cases	Constituent ratio (%)
Age	25∼35	34	12.78
	36∼45	60	22.56
	46∼55	96	36.09
	56∼65	56	21.05
	> 65	20	7.52

Gender	Male	74	27.82
	Female	192	71.18

Education level	Primary school	22	8.27
	Junior high school	66	24.81
	High school	66	24.81
	Junior college	54	20.30
	Undergraduate	42	15.79
	Postgraduate	16	6.01

Marital status	Unmarried	12	4.51
	Married	250	93.98
	Divorce	2	0.75
	Widowhood	2	0.75

Monthly income (RMB)	< 1000	12	4.51
	1000∼	50	18.80
	3000∼	124	46.61
	5000∼	42	15.79
	8000∼	24	9.02
	> 10,000	14	5.27

Residency style	Living with spouse	226	84.96
	Living with children	26	9.77
	Living alone	12	4.51
	Living with parents	2	0.75

Medical payment method	Social health insurance	214	80.45
	Commercial medical insurance	38	14.29
	Completely self-funded	4	1.50
	Others	10	3.76

Surgical approach	Unilateral lobectomy	48	18.04
	Partial lobectomy of the gland	145	54.51
	Total excision	73	27.44

Disease duration	< 6 months	84	31.58
	6 months∼1 year	56	21.05
	1∼1.5 years	42	15.79
	1.5∼2 years	28	10.53
	Over 2 years	56	21.05

Complicated by other diseases	No	210	78.95
	1∼2 kinds	54	20.30
	≥ 3 kinds	2	0.75

Family medical history	Yes	62	23.31
	No	204	76.69

**Table 2:** Medication adherence scores in postoperative thyroid cancer patients according to the Morisky Medication Adherence Scale (MMAS-8).

Projects	Yes	No	Mean (SD)
1. Do you sometimes forget to take your medicine?	98 (36.84%)	168 (63.16%)	0.63 ± 0.48
2. People sometimes miss taking their medications for reasons other than forgetting. Thinking over the past 2 weeks, were there any days when you did not take your medicine?	40 (15.04%)	226 (84.96%)	0.85 ± 0.36
3. Have you ever cut back or stopped taking your medication without telling your doctor because you felt worse when you took it?	6 (2.26%)	260 (97.74%)	0.98 ± 0.15
4. When you travel or leave home, do you sometimes forget to bring along your medication?	94 (35.34%)	172 (64.66%)	0.65 ± 0.48
5. Did you take your medication yesterday?	262 (98.50%)	4 (1.50%)	0.98 ± 0.12
6. When you feel like your illness is under control, do you sometimes stop taking your medicine?	16 (6.01%)	250 (93.98%)	0.94 ± 0.24
7. Taking medication every day is a real inconvenience for some people. Do you ever feel hassled about sticking to your treatment plan?	160 (60.15%)	106 (39.85%)	0.40 ± 0.49
8. How often do you have difficulty remembering to take all your medications?			0.82 ± 0.16
Never	100 (37.59%)	—	—
Occasionally	138 (51.88%)	—	—
Sometimes	28 (10.53%)	—	—

**Table 3:** One-way analysis of medication adherence in postoperative thyroid cancer patients (*N* = 266).

Projects	Number of cases	Score	*T/F*	*p* value
*Gender*			3.777	0.053
Male	74	5.95 ± 1.89		
Female	192	6.36 ± 1.42		

*Age (years)*			2.932	0.021
25∼35	34	6.04 ± 1.64		
36∼45	60	6.41 ± 1.46		
46∼55	88	6.36 ± 1.54		
56∼65	64	6.38 ± 1.52		
> 65	20	5.18 ± 1.79		

*Education level*			96.266	0.027
Primary school	22	5.20 ± 1.99		
Junior high school	66	6.51 ± 1.46		
High school	66	6.30 ± 1.57		
Junior college	54	6.31 ± 1.33		
Undergraduate	42	6.48 ± 1.30		
Postgraduate and above	16	5.56 ± 2.19		

*Marital status*			0.280	0.840
Unmarried	12	5.96 ± 1.43		
Married	250	6.27 ± 1.59		
Divorce	2	5.75 ± 0.00		
Widowhood	2	5.75 ± 0.00		

*Monthly income (RMB)*			40.915	0.114
< 1000	12	5.04 ± 3.02		
1000∼	50	6.51 ± 1.23		
3000∼	124	6.42 ± 1.46		
5000∼	42	6.23 ± 1.45		
8000∼	24	5.71 ± 1.37		
> 10,000	14	5.75 ± 2.02		

*Residence style*			0.666	0.574
Living with spouse	226	6.20 ± 1.61		
Living with children	26	6.63 ± 1.24		
Living alone	12	6.33 ± 1.63		
Living with parents	2	5.75 ± 0.00		

*Medical payment method*			16.288	0.003
Social health insurance	214	6.51 ± 1.45		
Commercial medical insurance	38	5.09 ± 1.40		
Completely self-funded	4	5.38 ± 1.88		
Others	10	5.35 ± 2.30		

*Surgical approach*			135.960	< 0.001
Unilateral lobectomy	48	5.30 ± 1.84		
Partial lobectomy of the gland	145	6.58 ± 1.12		
Total excision	73	6.21 ± 1.89		

*Disease duration*			133.802	< 0.001
< 6 months	84	6.80 ± 1.14		
6 months∼1 year	56	6.52 ± 1.48		
1∼1.5 years	42	5.93 ± 1.73		
1.5∼2 years	28	5.13 ± 2.12		
Over 2 years	56	5.95 ± 1.41		

*Complicated by other diseases*			2.993	0.052
No	210	6.36 ± 1.42		
1∼2 kinds	54	5.79 ± 2.03		
≥ 3 kinds	2	6.75 ± 0.00		

*Family medical history*			3.597	0.059
Yes	62	6.51 ± 1.39		
No	204	6.17 ± 1.62		

**Table 4:** Results of multiple linear regression analysis of medication adherence in patients with postoperative thyroid cancer (*N* = 266).

Variables	*B*	SE	*β*	*t*	*p*	95% CI (OR)		Covariance statistics	
						Bader limit	Upper limit	Tolerances	VIF
Constant term	2.355	0.697	—	3.381	0.001	5.567	8.073	—	—
Disease duration	−1.009	0.311	−0.197	−3.241	0.001	−0.342	−0.085	0.838	1.193
Education level	0.062	0.335	0.054	2.378	0.040	−0.102	0.218	0.674	1.484
Self-efficacy for rational medication use	0.122	0.017	0.458	7.288	< 0.001	0.098	0.163	0.710	1.409

**Table 5:** STROBE statement—checklist of items that should be included in reports of cross-sectional studies.

	Item no.	Recommendation
Title and abstract	1	(a) Indicate the study's design with a commonly used term in the title or the abstract
		(b) Provide in the abstract an informative and balanced summary of what was done and what was found

*Introduction*		
Background/rationale	2	Explain the scientific background and rationale for the investigation being reported
Objectives	3	State specific objectives, including any prespecified hypotheses

*Methods*		
Study design	4	Present key elements of study design early in the paper
Setting	5	Describe the setting, locations and relevant dates, including periods of recruitment, exposure, follow-up and data collection
Participants	6	(a) Give the eligibility criteria and the sources and methods of selection of participants
Variables	7	Clearly define all outcomes, exposures, predictors, potential confounders and effect modifiers. Give diagnostic criteria, if applicable
Data sources/measurement	8^∗^	For each variable of interest, give sources of data and details of methods of assessment (measurement). Describe comparability of assessment methods if there is more than one group
Bias	9	Describe any efforts to address potential sources of bias
Study size	10	Explain how the study size was arrived at
Quantitative variables	11	Explain how quantitative variables were handled in the analyses. If applicable, describe which groupings were chosen and why
Statistical methods	12	(a) Describe all statistical methods, including those used to control for confounding
		(b) Describe any methods used to examine subgroups and interactions
		(c) Explain how missing data were addressed
		(d) If applicable, describe analytical methods taking account of sampling strategy
		(e) Describe any sensitivity analyses

*Results*		
Participants	13^∗^	(a) Report numbers of individuals at each stage of study—e.g., numbers potentially eligible, examined for eligibility, confirmed eligible, included in the study, completing follow-up and analysed
		(b) Give reasons for nonparticipation at each stage
		(c) Consider use of a flow diagram
Descriptive data	14^∗^	(a) Give characteristics of study participants (e.g., demographic, clinical and social) and information on exposures and potential confounders
		(b) Indicate number of participants with missing data for each variable of interest
Outcome data	15^∗^	Report numbers of outcome events or summary measures
Main results	16	(a) Give unadjusted estimates and, if applicable, confounder-adjusted estimates and their precision (e.g., 95% confidence interval). Make clear which confounders were adjusted for and why they were included
		(b) Report category boundaries when continuous variables were categorized
		(c) If relevant, consider translating estimates of relative risk into absolute risk for a meaningful time period
Other analyses	17	Report other analyses done—e.g., analyses of subgroups and interactions and sensitivity analyses

*Discussion*		
Key results	18	Summarise key results with reference to study objectives
Limitations	19	Discuss limitations of the study, taking into account sources of potential bias or imprecision. Discuss both direction and magnitude of any potential bias
Interpretation	20	Give a cautious overall interpretation of results considering objectives, limitations, multiplicity of analyses, results from similar studies and other relevant evidence
Generalisability	21	Discuss the generalisability (external validity) of the study results

*Other information*		
Funding	22	Give the source of funding and the role of the funders for the present study and, if applicable, for the original study on which the present article is based

*Note:* An Explanation and Elaboration article discusses each checklist item and gives methodological background and published examples of transparent reporting. The STROBE checklist is best used in conjunction with this article (freely available on the Web sites of PLoS Medicine at https://www.plosmedicine.org/, Annals of Internal Medicine at https://www.annals.org/ and Epidemiology at https://www.epidem.com/). Information on the STROBE Initiative is available at https://www.strobe-statement.org.

^∗^Give information separately for exposed and unexposed groups.

## Data Availability

The data used to support the results of this study were restricted by the Ethics Committee of the Affiliated Hospital of Qingdao University to protect patient privacy. Data are available from Wen Li, liwencoco95@163.com, for researchers who meet the criteria for access to confidential data.

## Appendix A

STROBE Statement—Checklist of items that should be included in reports of cross-sectional studies is presented in.
